# Performance of the Two-Source Energy Balance (TSEB) Model as a Tool for Monitoring the Response of Durum Wheat to Drought by High-Throughput Field Phenotyping

**DOI:** 10.3389/fpls.2021.658357

**Published:** 2021-04-16

**Authors:** David Gómez-Candón, Joaquim Bellvert, Conxita Royo

**Affiliations:** ^1^Efficient Use of Water in Agriculture Program, Institute of Agrifood Research and Technology (IRTA), Fruitcentre, PCiTAL, Parc Científic i Tecnològic Agroalimentari de Gardeny, Lleida, Spain; ^2^Sustainable Field Crops Program, Institute of Agrifood Research and Technology (IRTA), Lleida, Spain

**Keywords:** transpiration, remote sensing, plant height, yield, grain number, grain weight, LAI, UAV

## Abstract

The current lack of efficient methods for high throughput field phenotyping is a constraint on the goal of increasing durum wheat yields. This study illustrates a comprehensive methodology for phenotyping this crop's water use through the use of the two-source energy balance (TSEB) model employing very high resolution imagery. An unmanned aerial vehicle (UAV) equipped with multispectral and thermal cameras was used to phenotype 19 durum wheat cultivars grown under three contrasting irrigation treatments matching crop evapotranspiration levels (ETc): 100%ETc treatment meeting all crop water requirements (450 mm), 50%ETc treatment meeting half of them (285 mm), and a rainfed treatment (122 mm). Yield reductions of 18.3 and 48.0% were recorded in the 50%ETc and rainfed treatments, respectively, in comparison with the 100%ETc treatment. UAV flights were carried out during jointing (April 4th), anthesis (April 30th), and grain-filling (May 22nd). Remotely-sensed data were used to estimate: (1) plant height from a digital surface model (H, *R*^2^ = 0.95, RMSE = 0.18m), (2) leaf area index from multispectral vegetation indices (LAI, *R*^2^ = 0.78, RMSE = 0.63), and (3) actual evapotranspiration (ETa) and transpiration (T) through the TSEB model (*R*^2^ = 0.50, RMSE = 0.24 mm/h). Compared with ground measurements, the four traits estimated at grain-filling provided a good prediction of days from sowing to heading (DH, *r* = 0.58–0.86), to anthesis (DA, *r* = 0.59–0.85) and to maturity (*r* = 0.67–0.95), grain-filling duration (GFD, *r* = 0.54–0.74), plant height (*r* = 0.62–0.69), number of grains per spike (NGS, *r* = 0.41–0.64), and thousand kernel weight (TKW, *r* = 0.37–0.42). The best trait to estimate yield, DH, DA, and GFD was ETa at anthesis or during grain filling. Better forecasts for yield-related traits were recorded in the irrigated treatments than in the rainfed one. These results show a promising perspective in the use of energy balance models for the phenotyping of large numbers of durum wheat genotypes under Mediterranean conditions.

## Introduction

Wheat is a staple food for humans, providing 18% of the daily human intake of calories and 20% of protein (http://www.fao.org/faostat/). Durum wheat (*Triticum turgidum* L. subsp. *durum* [Desf.] Husn) represents about 6% of a global wheat production of about 740 million tons per year (FAO, 2017). Wheat production per unit area needs to double by 2050 to meet the projected food demand of a global population forecast to be 9.22 billion. Achieving this objective is a significant challenge that will require increasing the current global yield increase rate of 1.3–2.4% y^−1^ (Ray et al., 2013), whilst at the same time minimizing the use of resources and the environmental impact (Tilman et al., 2011; Lal, 2016). Besides, in the current scenario of global climate change, the success of sustainable agriculture in many regions of the world is totally reliant on water availability. The Mediterranean region –the largest durum wheat producing area worldwide, the largest consumer of durum wheat products and the most important import market–, is one of the most sensitive to the effects of climate change, with projections forecasting a precipitation decrease of 4–27% during the cropping season (Flato et al., 2013). The development of high-yielding cultivars adapted to water-limited conditions is therefore critical to guarantee food security.

There is a general agreement that yield increases can only be achieved by improving the efficiency of large-scale breeding programs, particularly for suboptimal environments (Moshelion and Altman, 2015). One of the major challenges facing breeding programs centered on drought-prone areas is to develop tools capable of quantifying the actual water use of plants under different water regimes. The development of wheat varieties with improved water use efficiency (WUE, yield as a function of water used in transpiration) is seen as a way to increase yield in rainfed environments (Condon, 2004; Condon and Maxwell, 2014). The major challenge for fast genetic progress is to connect genetic variants (genotype) to their expression in observable traits (phenotype), and to predict plant phenotypes from genetic information (Cobb et al., 2013). The enormous advances in the genome sequencing of plants are providing massive genomic datasets, but the lack of efficient methods to rapidly collect large volumes of high quality phenotypic data has become a bottleneck in genomics-assisted breeding (White et al., 2012). Until now, given the complexity of measuring actual transpiration or water status in a large number of plots under field conditions, the difficulty of measuring the phenotypic response of plants to water use constraints has limited the goal of higher yields in breeding programs. Given this difficulty, evaluations of plant transpiration have relied mostly on surrogate traits, although this has most likely resulted in over-dependence on the surrogates (Vadez et al., 2014). Moreover, traditional phenotyping in germplasm evaluation activities under field conditions requires substantial investments in time, labor, and cost.

There is growing scientific interest in the application of remote sensing for high throughput phenotyping (HTP), particularly in breeding and germplasm evaluation activities (Furbank and Tester, 2011, Fiorani and Schurr, 2013; Walter et al., 2015). HTP through remote sensing allows the assessment of plant phenotypes on a scale and with a level of precision and speed that are unattainable with traditional methods (Dhondt et al., 2013). Numerous studies have used either RGB, fluorescent, thermal, hyperspectral, or 3D imaging to estimate morphological traits, biomass, plant growth, yield, water status, canopy temperature, or disease symptoms in many breeding programs and crops (Deery et al., 2014; Haghighattalab et al., 2016; Watanabe et al., 2017; Yang et al., 2017, 2020; Sagan et al., 2019). In addition, crop growth rates and spatial mapping of crop height variations have been obtained in wheat at field scale, as well as in individual plots, from images obtained with an RGB camera mounted on an unmanned aerial vehicle (UAV) (Holman et al., 2016). Madec et al. (2017) obtained a reliable assessment of the height of wheat plants with a digital camera with a 6,000–4,000 pixel sensor mounted on a hexacopter. Shi et al. (2016) developed empirical models to estimate the leaf area index (LAI) and percent canopy cover of winter wheat. Bendig et al. (2014) estimated fresh and dry above-ground biomass in barley from RGB images captured from a small UAV. Chapman et al. (2014) estimated crop lodging in wheat plots of a breeding program from images taken by cameras mounted on a customized robotic helicopter. Detailed reviews on remote sensing tools and platforms available for HTP in a plant breeding context can be found in Araus and Cairns (2014) and Araus et al. (2018).

Water status has been assessed in different crops by HTP thermography (Costa et al., 2013; Leroux et al., 2016; Perich et al., 2019). In many studies, different approaches have been used to calculate the so-called crop water stress index (CWSI) (Jackson et al., 1981; Jones, 1999; Gonzalez-Dugo et al., 2015). However, when the CWSI is calculated either empirically through non-water stress baselines or with reference panels, comparison between cultivars can only be achieved in a relative way. This is because the CWSI depends, among other factors, on the stomatal response to the vapor pressure deficit (VPD), which varies between cultivars and crop developmental stages. Therefore, it is too complex to determine this response for large collections of cultivars. Surface energy balance (SEB) models have also been widely used for assessing the actual evapotranspiration and water status of many crops at different scales, mostly using satellite imagery (Bastiaanssen et al., 1998; Allen et al., 2007; McShane et al., 2017). Among the different SEB models, the two-source energy balance (TSEB) modeling scheme allows the estimation of transpiration and evaporation separately (Norman et al., 1995). However, if very high resolution thermal imagery is available, in which case it is possible to directly retrieve soil (T_soil_) and canopy (T_c_) surface temperatures, the model can also be used, obtaining in some cases higher accuracies (Nieto et al., 2018; Bellvert et al., 2020). As far as we are aware, only Bellvert et al. (2021) have used this model to date for field-based phenotyping (of a collection of almond rootstocks in their work), but this present work is the first to evaluate the feasibility of TSEB in a set of durum wheat cultivars.

The primary objective of this research was to determine the suitability of using the TSEB model for the assessment of actual evapotranspiration and its components in a collection of spring durum wheat cultivars grown under contrasting water regimes in a Mediterranean environment. The specific objectives were: (1) to quantify the yield penalty caused by a reduction of water availability, (2) to determine the crop growing stage most suitable for assessing important agronomic traits through remote sensing images, and (3) to identify the agronomic traits that can be reliably assessed by remote sensing images and the best performing indicators for them.

## Materials and Methods

### Experimental Setup

**Figure 1:**
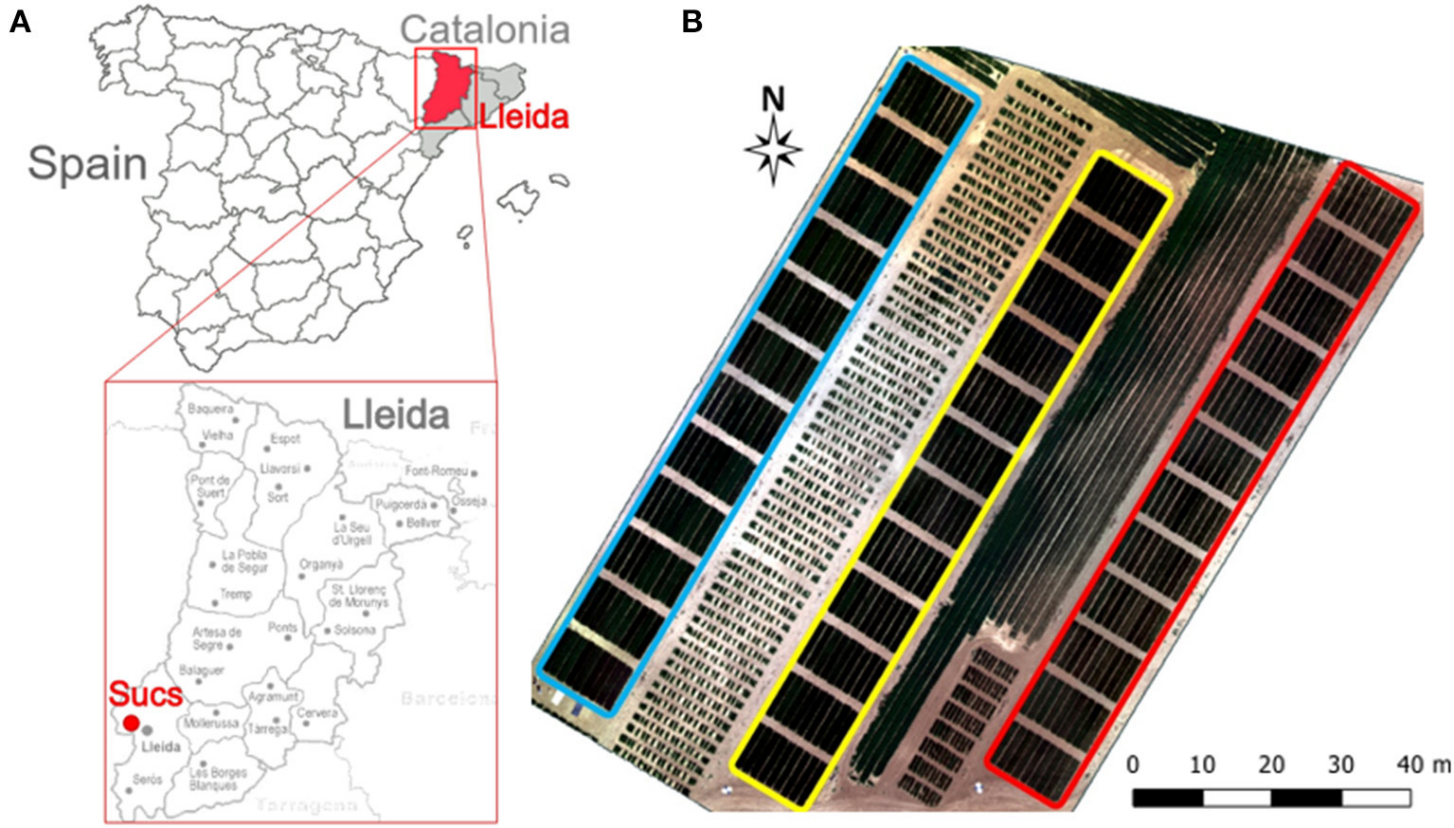
**(A)** Study site location; **(B)** Layout of the field experiment showing the three irrigation treatments: 100%ETc (blue), 50%ETc (yellow), and rainfed (red).

The field experiment was conducted at Sucs, Spain (41°41′49″N, 0°25′46″E, 285 m elevation) during the 2018–2019 growing season. The site has a typical Mediterranean climate, with a rainfall and reference evapotranspiration (ET_0_) of 177 and 603 mm, respectively, during the growing season. Soil has a fine-loamy texture with a field capacity of 27% and wilting point of 13% as calculated from the Saxton's soil hydraulic calculator (Saxton et al., 1986). Fourteen durum wheat (*Triticum turgidum* ssp. *durum*) commercial varieties (Anvergur, Athoris, Burgos, Calero, Carpio, Claudio, Don Ricardo, Don Sebastián, Eunoble, Euroduro, Grador, Iberus, Sculptur, and Tussur) and five inbred lines from the IRTA durum wheat breeding program (05D278, 07D057, 08D010, 09D066, 09D069) were evaluated under three contrasting irrigation treatments. Irrigation treatments were as follows: (i) 100%ETc, irrigated 100% of the seasonal crop evapotranspiration (ETc), (ii) 50%ETc, irrigated 50% of seasonal ETc, and (iii) Rainfed, which was not irrigated (Figure 1). In each irrigation treatment, genotypes were planted following an incomplete block design with four replications and plots of 9.6 m^2^ (eight rows 8 m long and 0.15 m apart). Sowing was carried out on December 4th 2018 at a density of 450 seeds/m^2^. Due to the low precipitation received from December to February (29 mm) all plots were evenly irrigated on March 1st with 20 mm to guarantee the plants' survival. Irrigation was scheduled on a weekly basis and water was applied during 2–3 days of the week. Sprinklers were installed in a grid of 18 × 18 m and water flow discharge was 7.8 l/h/m^2^ for the 100%ETc and 3.9 l/h/m^2^ for the 50%ETc treatments. Weekly irrigation was scheduled following a water balance model (Allen et al., 1998). ETc was calculated as a product of the Penman-Monteith ET_0_ (Allen et al., 1998) and crop coefficients (Kc). The used crop coefficients were derived from FAO-56 (Allen et al., 1998), and started from 0.7 at the vegetative growth stage to 1.07 at the beginning of the mid-season stage. During the late season (from June 11th), the Kc decreased and reached a value of 0.6. In addition, 0.8 was used as a coefficient of efficiency of the sprinkler irrigation system (Savva et al., 2001). Meteorological data was gathered from an automated weather station belonging to Catalonia's official network of meteorological stations (SMC, www.ruralcat.net/web/guest/agrometeo), which is located around 3 km from the study site. The amount of water applied through irrigation in each treatment during the entire growing season was also measured with digital water meters (CZ2000-3M, Contazara, Zaragoza, Spain). Before sowing, the field was fertilized with 162 u P_2_O_5_ and 360 u K_2_O ha^−1^ and top dressed twice with ammonium nitro-sulfate at rates of 118 kg N/ha at the end of tillering and 50 kg N/ha at mid-jointing. The field was maintained free of weeds, diseases and pests by chemical treatments.

### Image Acquisition Campaign

**Figure 2:**
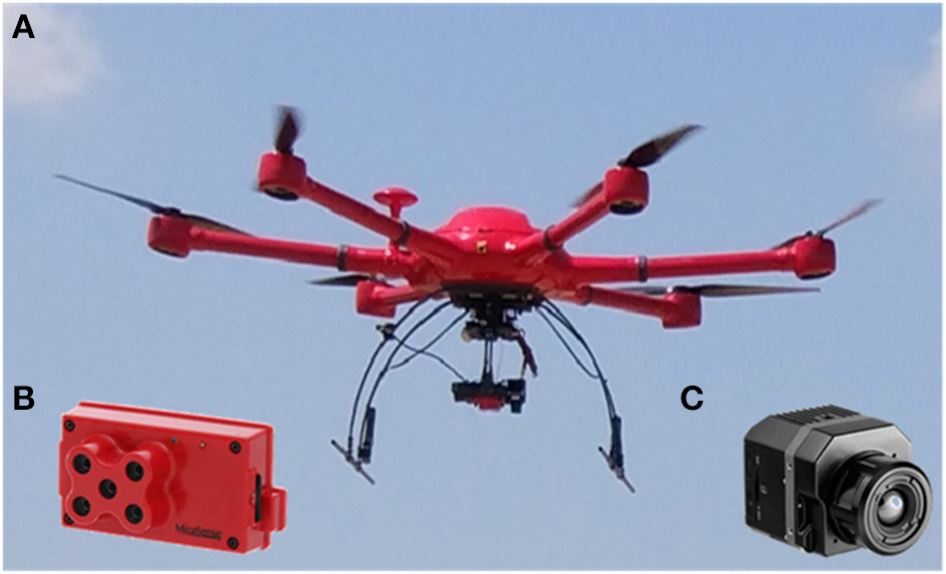
Unmanned aerial vehicle (UAV) and cameras used in the study. **(A)** UAV, **(B)** Multispectral Micasense Rededge, and **(C)** Thermal Flir Vue Pro cameras.

**Figure 3:**
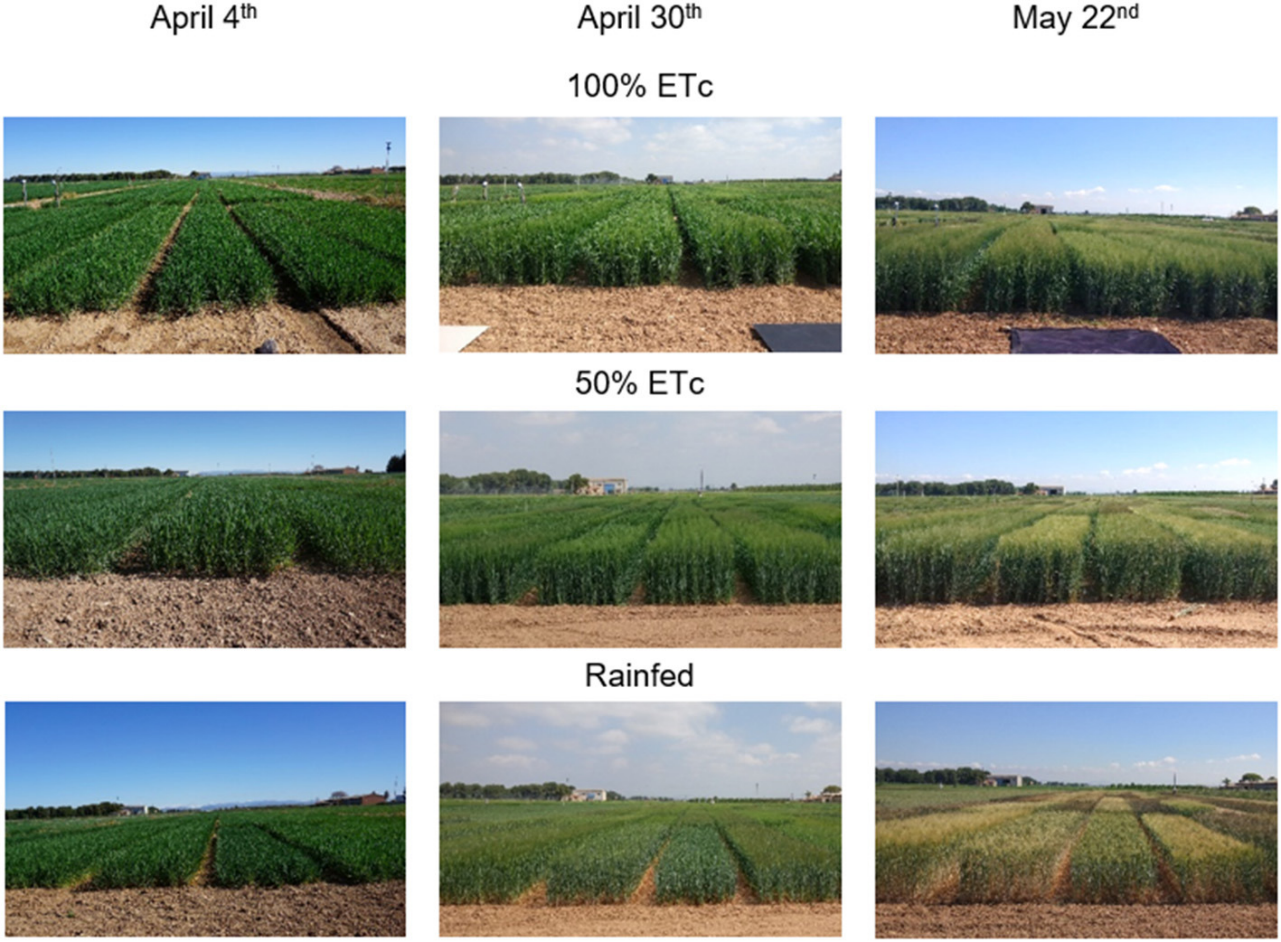
Pictures of each irrigation treatment for the different dates of image acquisition (April 4th, April 30th, and May 22nd).

Images were acquired with the Cóndor UAV hexacopter (Dronetools, https://www.dronetools.es/) (Figure 2) on April 4th (121DAS, days after sowing), April 30th (147DAS), and May 22nd (169DAS) in 2019, coinciding with the crop developmental stages of mid-jointing, around anthesis and grain filling, respectively (Figure 3). The vapor pressure deficit (VPD) and air temperature (T_a_) at the moment of image acquisition were respectively, 5.5 KPa and 9.9°C for 121DAS, 12.0 KPa and 18.8°C for 147DAS, and 11.0 KPa and 19.7°C for 169DAS. Flights were always conducted in sunny conditions and with a wind speed below 12 m/s. The UAV was equipped with a multispectral and a thermal camera. The former was a Micasense RedEdge-M (Micasense, 1300 N Northlake Way, Seattle, USA), which has five spectral bands located at the wavelengths 475 ± 20 nm (blue), 560 ± 20 nm (green), 668 ± 10 nm (red), 717 ± 10 nm (red edge), and 840 ± 40 nm (near infrared), and a field of view (FOV) of 47.2°. The thermal camera was a FLIR Vue PRO (FLIR Systems, Wilsonville, OR, USA) with a resolution of 336 × 256 pixels and a 6.8 mm focal length, with a FOV of 45 × 35°. The spectral response was in the range of 7.5–13.5 μm. All flights were conducted at ~12:00 h solar time. The UAV flew over at a height of 50 m agl (above ground level), capturing images with a resolution of 0.02 and 0.10 m per pixel for the multispectral and thermal cameras, respectively. Flight planning had 80/60 frontal and side overlap, respectively. During image acquisition, *in situ* measurements were conducted for different targets in order to correct the atmospheric contribution to the signal. Temperature measurements were continuously recorded for hot and cold targets (black and white panels, bare soil, and vegetation) with a fixed IR-temperature sensor (Calex PC151LT-O, Pyrocouple series, Calex Electronics Limited, Bedfordshire, UK). The radiometric calibration of the multispectral sensor was conducted through an external incident light sensor that measured the irradiance levels of light at the same bands as the camera. In addition, *in situ* spectral measurements for ground calibration targets were performed using a Jaz spectrometer (Ocean Optics, Inc., Dunedin, FL, USA). The Jaz has a wavelength response from 200 to 1,100 nm and an optical resolution of 0.3 to 10.0 nm. During spectral collection, spectrometer calibration measurements were taken with a reference panel (white color Spectralon^TM^) and dark current before and after taking readings from radiometric calibration targets. Geometrical correction was conducted using five ground control points (GCP), and measuring the position in each with a handheld global positioning system (GPS) (Geo7x, Trimble GeoExplorer series, Sunnyvale, CA, USA). All images were mosaicked using the Agisoft Photoscan Professional version 1.6.2 (Agisoft LLC., St. Petersburg, Russia) software and geometrically and radiometrically terrain corrected with QGIS 3.4 (QGIS 3.4.15).

### Measurements of Agronomic Traits

Crop development was monitored on three replications per treatment on a twice-weekly basis from booting to record the following growth stages (Zadoks et al., 1974): GS55 (heading), GS65 (anthesis), and GS87 (physiological maturity). A plot was considered to have reached a given developmental stage when ~50% of the plants exhibited the stage-specific phenotypic characteristics. For each UAV flight date, on-ground key crop biophysical parameters were measured as follows. Plant height (PH, cm) was measured in three plants per plot of one replication for each irrigation treatment. PH was also measured at GS87 in three main stems per plot in three replications from the tillering node to the top of the spike, excluding the awns. The LAI was obtained on the same days and in the same plots using a portable linear ceptometer (AccuPAR model LP-80, Decagon Devices Inc., Pullman, WA, USA). Measurements were conducted from 12:00 to 15:00 h (local time) in one replicate of each irrigation treatment. In total, 63 plots were measured for each flight event. Photosynthetically active radiation (PAR) below the wheat was measured placing the ceptometer in a horizontal position at ground level and recording five PAR readings in each plot. A fixed tripod connected to the sensor allowed collection of the incident radiation above the plants. Then, the LAI calculator provided by AccuPAR-L80 (LAI-calculator, METER Group) was used to estimate LAI. Concomitant to image acquisition, in three leaves per plot of one replication in each irrigation treatment, leaf transpiration was also measured with an infrared gas analyzer (IRGA) (LI-7500, LI-COR Inc., Lincoln, NE). Plots were harvested mechanically at ripening and yield (kg/ha) expressed as dry weight. From a random sample of the plants contained in a 0.5-m-long stretch from a central row of each plot of three replications at ripening, the number of spikes/m^2^ (NSm^2^) and the number of grains/spike (NGS) were assessed. Thousand kernel weight (TKW) was estimated as the mean weight of three sets of 100 grains per plot. Grain filling rate (GFR, mg/day) was obtained as the quotient between grain dry weight and grain-filling duration (GFD) considered to be the number of days between anthesis and physiological maturity.

### Remotely-Sensed Estimates of Biophysical Traits

**Figure 4:**
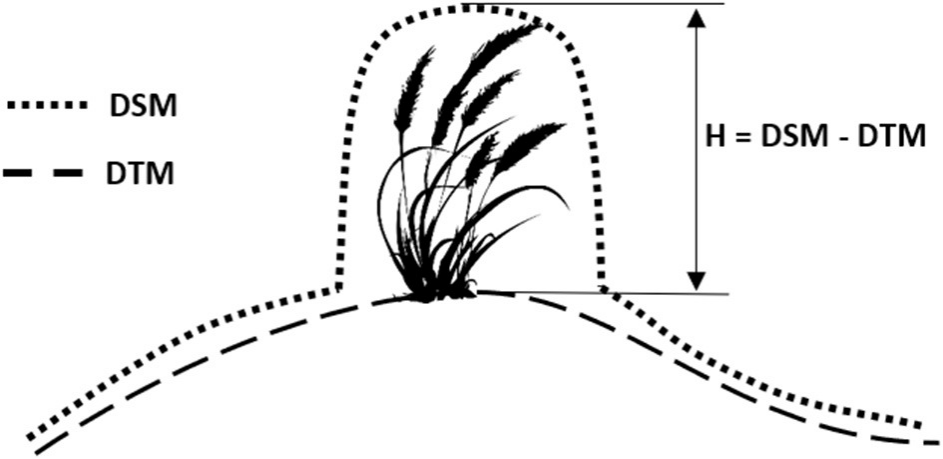
Schematic workflow used to estimate crop height. Digital terrain model (DTM) and digital surface model (DSM) obtained through automatic aerial triangulation. Plant height (H) estimated above ground surface.

The three-dimensional plant height (H) was estimated from the photogrammetric point cloud of multispectral images. The digital surface model (DSM) and the digital terrain model (DTM, bare soil surface devoid of plants) were both obtained through automatic aerial triangulation, bundle block adjustment, and camera calibration methods using the Agisoft PhotoScan version 1.6.2 (Agisoft, 2020; St. Petersburg, Russia) software. A classification of bare ground pixels was used to obtain the DTM of the field. Then, a raster corresponding to heights was obtained by subtracting the DTM from the DSM using the band math tool of the QGIS software (Figure 4). LAI was estimated from spectral vegetation indices. In particular, this study used the improved modified triangular vegetation index (MTVI_2_) (Yao et al., 2017), which was calculated as:

(1) $${\text{MTVI}}_{\text{2}}=\frac{\text{1}.\text{5}\left[\text{1}.\text{2}\left({\text{R}}_{\text{840}}-{\text{R}}_{\text{560}}\right)-\text{2}.\text{5}\left({\text{R}}_{\text{717}}-{\text{R}}_{\text{560}}\right)\right]}{\sqrt{{\left({\text{2R}}_{\text{840}}\text{+1}\right)}^{\text{2}}-\left({\text{6R}}_{\text{840}}-\text{5}\sqrt{{\text{R}}_{\text{717}}}\right)-\text{0}.\text{5}}}$$

The fractional vegetation cover (fc) of each plot was also calculated by adapting the equation proposed by Gutman and Ignatov (1998). Instead of the normalized difference vegetation index (NDVI), we used the MTVI_2_ due to its low saturation at high LAI values:

(2) $$\begin{array}{r} f_{c}=\frac{{\text{MTVI}2}_{i}-{MTVI2}_{soil}}{{\text{MTVI}2}_{veg}-{MTVI2}_{soil}} \end{array}$$

where

MTVI_2i_ corresponds to the value on the target plot;

MTVI_2soil_ corresponds to the value of bare soil; and

MTVI_2veg_ corresponds to the value of pure vegetation.

### Remotely-Sensed Estimates of Evapotranspiration

Actual crop evapotranspiration (ETa) and its partition components were retrieved from the two-source energy balance (TSEB) model (Norman et al., 1995; Kustas and Anderson, 2009). Two-source models partition the surface energy fluxes and the radiometric temperature (T_rad_) between nominal soil and canopy sources. The approach is therefore able to estimate canopy transpiration (T) and soil evaporation (E) separately. However, because direct measurements of canopy (T_c_) and soil (T_soil_) temperatures are rarely available with satellite imagery, in most applications these component temperatures are estimated in an iterative process in which it is first assumed that green canopy (expressed as the function of LAI that is green) transpires at a potential rate based on the Priestley-Taylor formulation (Priestley and Taylor, 1972). On the other hand, if very high resolution thermal imagery is available, it is possible to obtain T_s_ and T_c_ directly, without the need to compute an initial canopy transpiration (Nieto et al., 2018; Bellvert et al., 2021). In this study, T_soil_ and T_c_ were individually obtained for each plot from the thermal imagery. The model also requires other inputs such as plant height, LAI and f_c_, the retrieval of which has been described above. Meteorological inputs were obtained from Catalonia's official network of meteorological stations. For more information, the full python script is available online (https://github.com/hectornieto/pyTSEB, last accessed 20.08.2020) and additional details of the TSEB model are provided by Norman et al. (1995); Kustas and Norman (1999), and Nieto et al. (2018).

### Statistical Analyses

Analyses of variance (ANOVAs) were conducted following a split-plot design. Means were compared with a Tukey test at *P* < 0.05. Linear regression equations and Pearson correlation coefficients were used to analyze the relationship between variables.

## Results

### Effect of Irrigation Treatments on the Agronomic Performance of Durum Wheat

**Figure 5:**
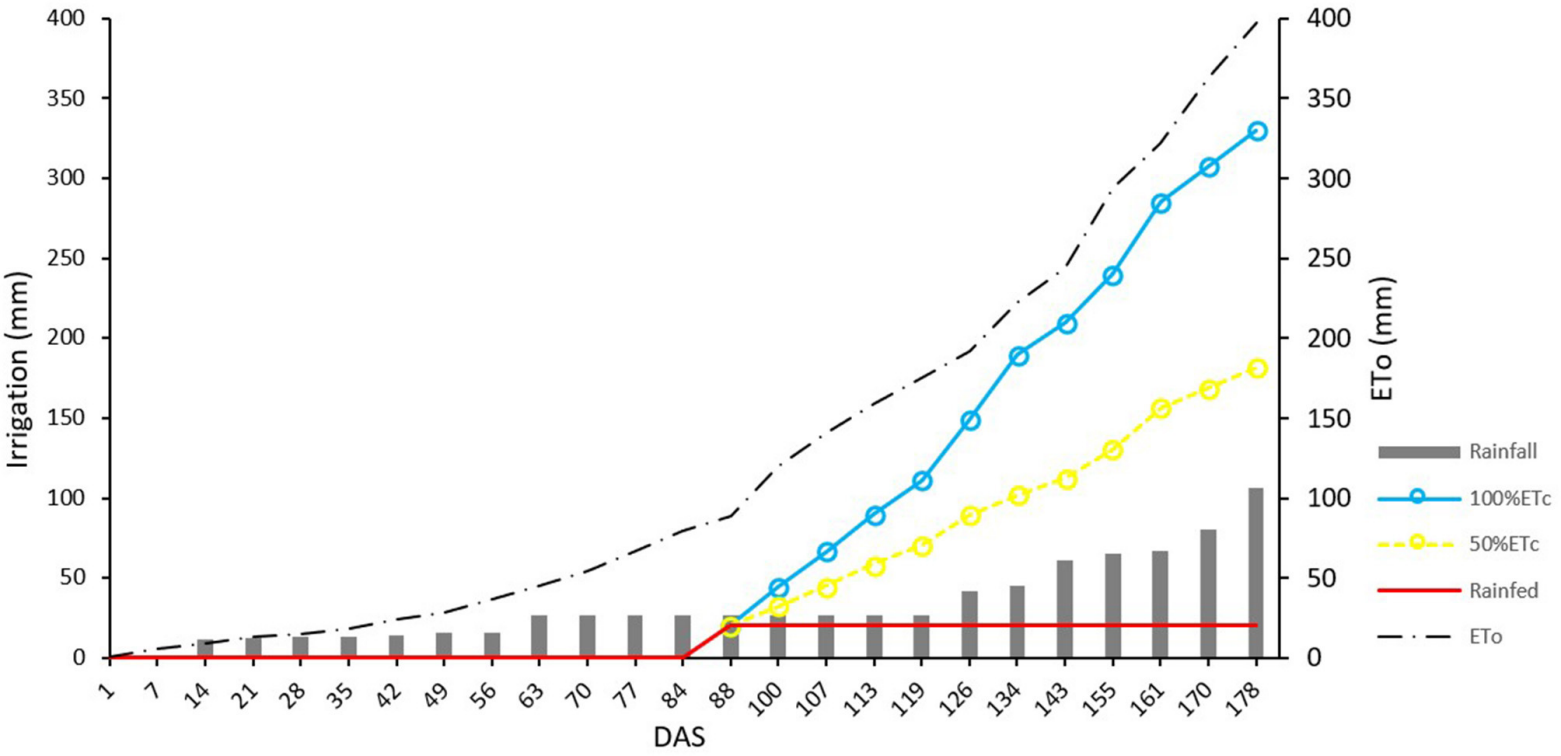
Amount of water applied, rainfall, and reference evapotranspiration (ET_0_) corresponding to the different irrigation treatments (100%ETc, 50%ETc, and rainfed) from March 2019 (87 DAS, days after sowing) to May 2020 (177 DAS).

The amount of irrigation water applied throughout the growing season in the 100%ETc and 50%ETc treatments was 340 and 180 mm, respectively (Figure 5). When also considering the rainfall from sowing to physiological maturity, the total amount of water received was 450, 285, and 122 mm for the 100%ETc, 50%ETc, and rainfed treatments, respectively. The ANOVAs showed statistically significant differences among irrigation treatments for all of the evaluated agronomic traits (Table 1). Yield ranged between 7,274 and 10,446 kg/ha in the 100%ETc, between 5,910 and 8,469 kg/ha in the 50%ETc and between 3,905 and 5,972 kg/ha in the rainfed treatments (Table 1 and Supplementary Table 1). The effect of the total amount of water applied on yield was huge, as the 50%ETc and rainfed treatments reduced yield on average by 18.3 and 48.0%, respectively, in comparison to the treatment meeting all crop water requirements. While the 50%ETc treatment did not diminish the NSm^2^, it did decrease the NGS and TKW. The absence of irrigation resulted in larger reductions in NGS than in NSm^2^ and TKW. The grain filling rate increased steadily as consequence of water shortage. Plant height was reduced 6.5 and 11.9% in the 50%ETc and rainfed treatments in comparison with 100%ETc.

**Table 1:** Mean values ± SE and ranges (between brackets) for yield and yield-related traits of the 19 durum wheat genotypes included in the study.

**Trait**	**Irrigation treatment**					
	**100% ETc**		**50% ETc**		**Rainfed**	
Grain yield (kg/ha)	9219 ± 184^a^	(7,274–10,446)	7534 ± 184^b^	(5,910–8,469)	4793 ± 120^c^	(3,905–5,972)
Number of spikes/m^2^ (NSm^2^)	501 ± 26.1^a^	(289–742)	523 ± 16.0^a^	(387–627)	452 ± 13.6^b^	(356–591)
Number of grains/spike (NGS)	42.1 ± 1.53^a^	(34.3–60.3)	35.8 ± 1.56^b^	(23.6–53.9)	27.9 ± 1.32^c^	(15.3–38.8)
Thousand kernel weight (TKW, g)	55.8 ± 0.69^a^	(45.5–64.8)	53.7 ± 1.01^b^	(44.9–61.3)	51.5 ± 0.78^c^	(45.1–58.4)
Grain filling rate (GFR, mg/day)	1.39 ± 0.04^c^	(1.08–1.64)	1.42 ± 0.03^b^	(1.11–1.67)	1.47 ± 0.03^a^	(1.24–1.68)
Plant height (PH, cm)	92 ± 1.44^a^	(80–102)	86 ± 1.29^b^	(73–95)	81 ± 1.01^c^	(72–92)
Days to heading (DH)	135 ± 0.55^a^	(130–138)	132 ± 0.52^b^	(127–136)	128 ± 0.43^c^	(124–130)
Days to anthesis (DA)	143 ± 0.66^a^	(138–147)	139 ± 0.54^b^	(135–143)	135 ± 0.28^c^	(133–137)
Days to maturity (DM)	183 ± 0.39^a^	(180–186)	177 ± 0.35^b^	(174–143)	170 ± 0.45^c^	(167–174)
Grain filling duration (GFD, days)	40 ± 0.41^a^	(37–43)	38 ± 0.41^b^	(35–40)	35 ± 0.38^c^	(32–38)

*Means within rows with different letters are significantly different for a Tukey test at P < 0.05*.

Reductions in the amount of water applied also significantly shortened the crop cycle (Table 1). In comparison with 100%ETc, the 50%ETc treatment resulted in decreases of 3 (2.2%), 4 (2.8%), and 6 (3.3%) days in the length of the periods from sowing to heading, anthesis, and maturity, respectively. The rainfed treatment additionally shortened between 4 and 7 days the periods needed to reach each of these growth stages. In consequence, a significant drop was observed in the duration of the grain-filling period.

### Remotely-Sensed Estimates of the Biophysical Parameters and Evapotranspiration Components

The ANOVA showed statistically significant differences between flights, irrigation treatments and their interaction (Table 2). This interaction was of a cross-over nature due to the opposite trend observed in the first flight compared with the second and third ones, as shown in Table 3. Genotypes also differed for all remotely-sensed traits. The interactions of genotype with flight date and irrigation treatment were significant with the exception of the flight date × genotype interaction for H and ETa (Table 2).

**Table 2:** *P*-values of the ANOVAs for the traits estimated through remote sensing.

**Source of variation**	**D.F**.	**ETa**	**T**	**H**	**LAI**
Flight date	2	<0.0001	<0.0001	<0.0001	<0.0001
Irrigation treatment	2	<0.0001	<0.0001	<0.0001	<0.0001
Error a	6				
Flight date* Irrigation treatment	4	<0.0001	<0.0001	<0.0001	<0.0001
Error b	12				
Genotype	18	<0.0001	<0.0001	<0.0001	<0.0001
Flight date × Genotype	36	ns	0.0127	ns	0.0453
Irrigation treatment × Genotype	36	0.0005	0.0479	0.0124	0.0018
Residual	396				
Total	512				

*ETa, actual evapotranspiration; T, actual transpiration; H, estimated plant height*.

**Table 3:** Mean values ± SE for LAI, daily evapotranspiration (ETa), and daily transpiration (T) assessed by remote sensing imagery for each flight date and for each water input treatment.

**Irrigation treatment**	**April 4th**		**April 30th**		**May 22nd**	
	**Mean**	**Range**	**Mean**	**Range**	**Mean**	**Range**
**LAI**						
100%ETc	1.66 ± 0.03^c^	1.24–2.18	3.87 ± 0.05^a^	2.46–5.11	3.80 ± 0.07^a^	2.10–5.29
50%ETc	1.84 ± 0.02^b^	1.47–2.37	3.23 ± 0.05^b^	2.48–4.21	2.66 ± 0.06^b^	1.72–3.73
Rainfed	2.03 ± 0.04^a^	1.11–2.61	2.05 ± 0.04^c^	1.33–2.98	1.01 ± 0.03^b^	0.51–1.72
**ET**_**a**_ **(mm/day)**						
100%ETc	4.88 ± 0.04^c^	4.11–5.77	6.80 ± 0.03^a^	6.00–7.22	7.15 ± 0.07^a^	4.79–7.66
50%ETc	5.41 ± 0.03^b^	4.72–6.04	6.41 ± 0.04^a^	5.43–7.03	5.53 ± 0.09^b^	3.49–7.15
Rainfed	5.66 ± 0.06^a^	4.63–6.95	4.74 ± 0.08^b^	3.05–5.85	3.78 ± 0.03^c^	3.15–4.69
**T (mm/day)**						
100%ETc	2.45 ± 0.02^c^	2.06–2.89	4.45 ± 0.03^a^	3.98–5.01	5.47 ± 0.06^a^	3.90–6.68
50%ETc	2.56 ± 0.02^b^	2.23–2.92	4.49 ± 0.03^a^	3.93–5.03	4.55 ± 0.08^b^	3.38–6.30
Rainfed	2.67 ± 0.03^a^	1.86–3.03	3.73 ± 0.06^b^	2.68–4.89	2.06 ± 0.05^c^	1.01–3.30

*Means within columns and trait with different letters are significantly different for a Tukey test at P < 0.05*.

**Figure 6:**
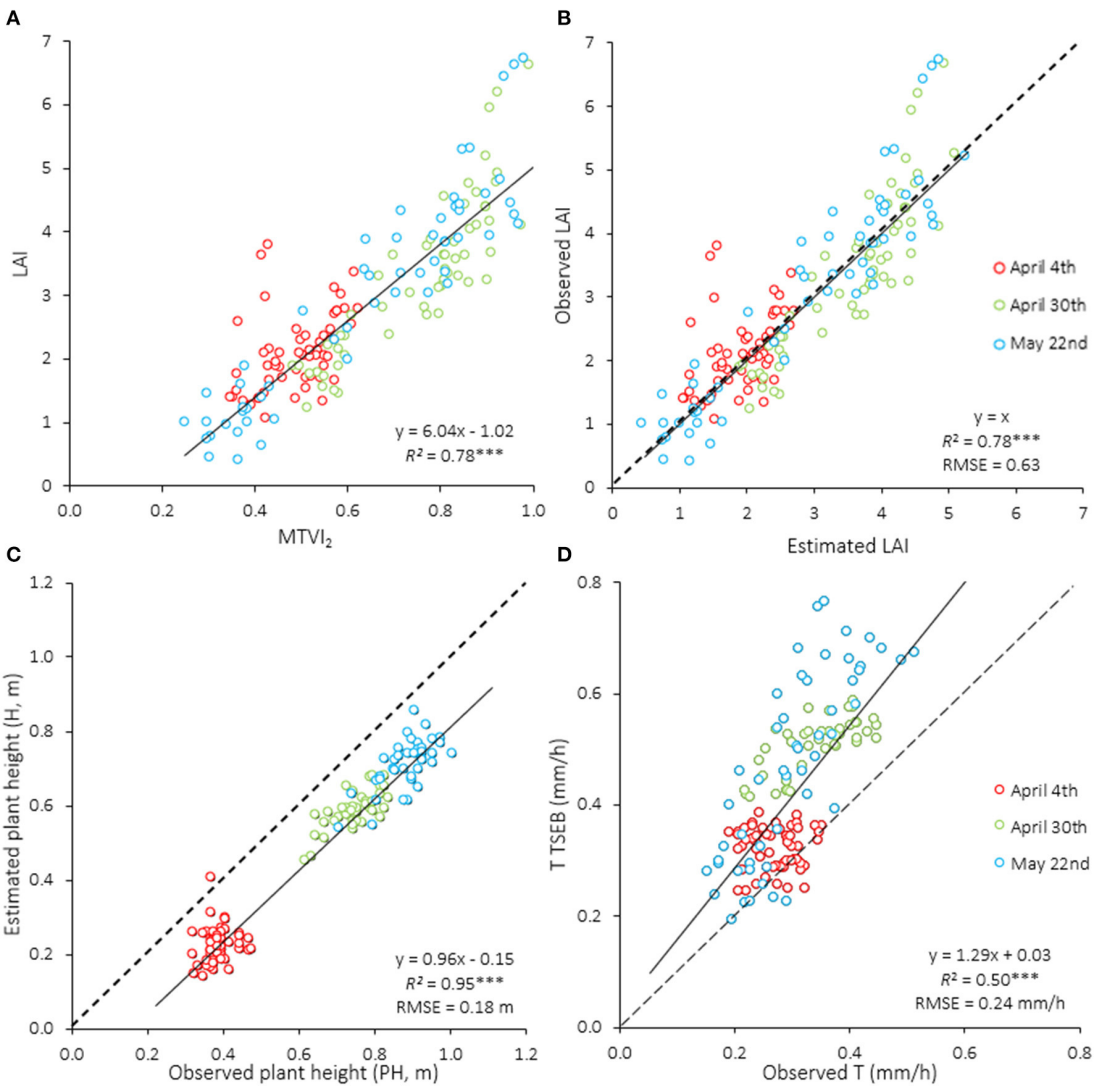
Relationships between **(A)** leaf area index (LAI) and the MTVI_2_ spectral vegetation index, **(B)** observed and estimated LAI by the MTVI_2_, **(C)** observed and estimated plant height by the photogrammetric 3D point cloud, and **(D)** observed leaf transpiration with the IRGA device and estimated plant transpiration (T) through the TSEB model. Different colors indicate different dates of image acquisition (*n* = 171). RMSE, root mean square error. ***P < 0.001; **P < 0.01; *P < 0.05.

The MTVI_2_ vegetation index (VI) was linearly related with LAI when aggregating data from the three flight dates (*R*^2^ = 0.78, *P* < 0.001, Figure 6A). Also, this regression was significant for each specific date, with *R*^2^ values of 0.20, 0.77, and 0.87 for April 4th, April 30th, and May 22nd, respectively. The one-to-one relationship between observed and estimated LAI showed an RMSE of 0.63 (Figure 6B). Average remotely-sensed estimated LAI values significantly increased from April 4th to April 30th, but slightly decreased at the third acquisition date (May 22nd) (Table 3). Differences in LAI between irrigation treatments were also significant for all image acquisition dates (*P* < 0.001). In contrast with the values observed for flights conducted at anthesis (April 30th) and grain-filling (May 22nd), the LAI values of the rainfed treatment at the jointing stage (April 4th) were the highest. Estimates of plant height through remote sensing were significant, with *R*^2^ of 0.95 and RMSE of 0.18 m when aggregating data from the three dates (Figure 6C). Averaged observed values of PH ranged from 0.40 to 0.96 m, respectively, for the first (April 4th) and last (May 22nd) flights. In all dates, the remotely-sensed assessments underestimated the actual PH. Estimates of canopy transpiration (T) obtained through the TSEB model were compared against those measured at leaf level. The regression obtained aggregating data from the three dates was significant (*R*^2^ = 0.50, *P* < 0.001) with an RMSE of 0.24 mm/h (Figure 6D). Differences in ETa and T between irrigation treatments were also significant for all dates (Table 3). Similarly to LAI, the highest and lowest values of ETa and T were respectively identified in the 100%ETc and rainfed treatments, with the partial exception of the flight conducted on April 4th (jointing stage), when the values were inverted.

### Relationships Between Agronomic and Remotely-Sensed Traits

**Figure 7:**
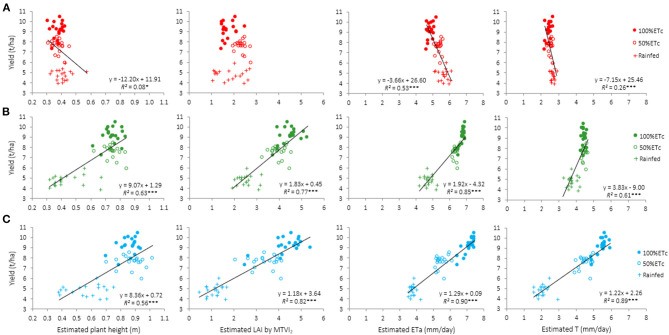
Relationships between estimated plant height, LAI, actual evapotranspiration (ETa), and transpiration (T) assessed from remote sensing imagery and yield for the three irrigation treatments (full circles, 100%ETc; empty circles 50%ETc; +, Rainfed) for each image acquisition event: **(A)** April 4th (jointing), **(B)** April 30th (anthesis), and **(C)** May 22nd (grain filling). Each point represents the mean value of a genotype across replications (*n* = 57). ***P < 0.001; **P < 0.01; *P < 0.05.

Regression analyses were carried out using the aggregated yield of the three irrigation treatments as dependent variable and each of the four traits assessed by remote sensing in each flight event as explanatory ones (Figure 7). The results show that LAI, estimated at jointing, could not predict yield. However, the relationships between yield and H, ETa, and T were negative and statistically significant in this first flight (Figure 7A). Significant and positive relationships were obtained between yield and remotely-sensed estimated traits on the other two image acquisition dates. With the exception of H, *R*^2^ tended to be slightly higher on the last date (May 22nd), accounting for between 82 and 90% of yield variability (Figure 7C). ETa was the parameter which showed the highest *R*^2^ with yield for the last two image acquisition dates. Although the *R*^2^ of the yield vs. T regressions were also high, the values were slightly lower in comparison with those obtained between yield and ETa.

**Figure 8:**
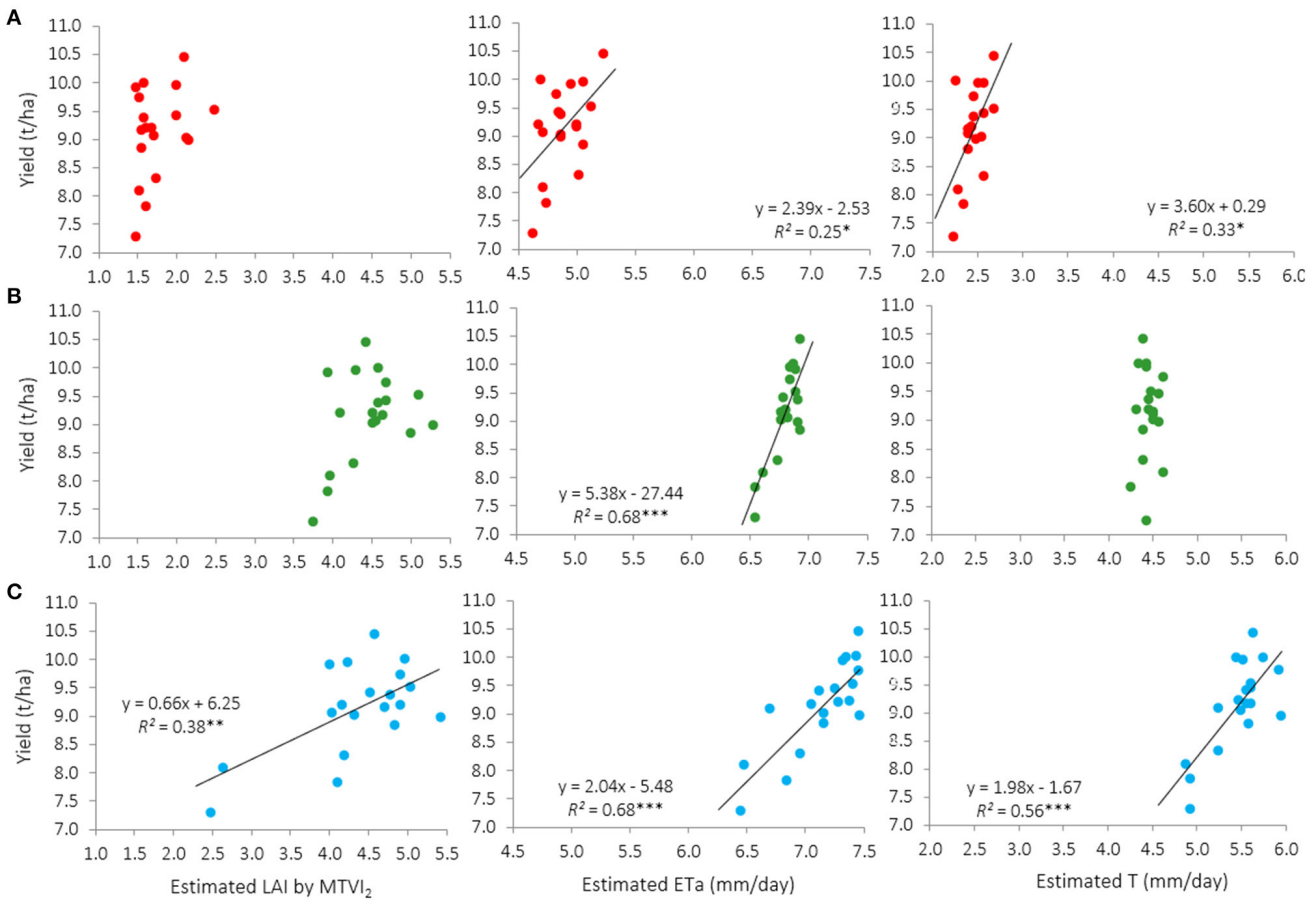
Relationships between estimated LAI, actual evapotranspiration (ETa), and transpiration (T) assessed from remote sensing images and yield for the 100%ETc treatment for each image acquisition event: **(A)** April 4th (jointing), **(B)** April 30th (anthesis), and **(C)** May 22nd (grain filling). Each point represents the mean value of a genotype across replications (*n* = 19). Relationships involving predicted plant height are not shown because *P* > 0.05 in the three image acquisition dates. ***P < 0.001; **P < 0.01; *P < 0.05.

For a deeper analysis, the same relationships were examined for each irrigation treatment separately. Significant associations (*P* < 0.05) between remotely-sensed traits and yield were only found for the 100%ETc treatment (Figure 8). The non-significant relationships of other irrigation treatments were probably due to the lower range of yield values obtained in them, as shown in Table 1. In addition, the relationships between H and yield in the 100%ETc treatment were also not significant (data not shown). The accuracy of fitting yield to LAI, ETa, and T varied between dates (Figure 8). The only trait significantly related with yield in the three dates was ETa, which at anthesis and grain filling accounted for 68% of yield variations (Figures 8B,C). On the other hand, the relationship between T and yield was also slightly lower in comparison with ETa.

Most of the relationships between traits estimated through remote sensing (ETa, T, H, and LAI) and the agronomic traits other than yield were statistically significant when the data of the three irrigation treatments were aggregated for the analyses (Table 4). The second and third flights led to the largest number of significant and positive correlation coefficients, in contrast with the negative associations obtained in the first flight, as observed previously for the relationships with yield. The largest Pearson correlation coefficients (*r* > 0.80, *P* < 0.001) corresponded to the relationships between ETa, T, and LAI with DH, DA, and DM, particularly during the third image acquisition data (grain filling) (Table 4). Correlation coefficients between the four remotely-sensed traits and PH were also positive and significant in the two later flights. For the yield components, the largest correlation coefficients appeared for the relationships between ETa, T, and LAI with NGS, and between H and both PH and TKW. Traits assessed from remote sensing could not properly estimate GFR when data of the three irrigation treatments were analyzed at once. Strong relationships were observed between remotely-sensed and phenological traits (Table 4).

**Table 4:** Pearson correlation coefficients (*r*) for the relationships between traits assessed through remote sensing and agronomic traits other than yield for each image acquisition event across irrigation treatments (*n* = 57).

**Trait**	**April 4th**				**April 30th**				**May 22nd**			
	**ETa**	**T**	**H**	**LAI**	**ETa**	**T**	**H**	**LAI**	**ETa**	**T**	**H**	**LAI**
Number of spikes/m^2^ (NSm^2^)	−0.01	0.08	−0.02	0.25	0.36**	0.38**	0.30*	0.37**	0.28*	0.35**	0.30*	0.35**
Number of grains/spike (NGS)	−0.74***	−0.68***	−0.44***	−0.20	0.59***	0.49***	0.41**	0.54***	0.64***	0.63***	0.41**	0.55***
Thousand kernel weight (TKW)	−0.23	−0.14	0.13	0.11	0.38**	0.27*	0.50***	0.37**	0.39**	0.37**	0.42**	0.38**
Grain filling rate (GFR)	0.28*	0.28**	0.39**	0.07	−0.19	−0.20	−0.03	−0.16	−0.19	−0.18	−0.04	−0.13
Plant height (PH)	−0.40**	−0.20	−0.01	0.11	0.66***	0.55***	0.64***	0.64***	0.69***	0.67***	0.62***	0.65***
Days to heading (DH)	0.65***	−0.48***	−0.24	0.03	0.80***	0.71***	0.66***	0.82***	0.86***	0.85***	0.58***	0.82***
Days to anthesis (DA)	−0.67***	−0.49***	−0.22	−0.02	0.78***	0.67***	0.62***	0.81***	0.85***	0.83***	0.59***	0.81***
Days to maturity (DM)	−0.80***	−0.63***	−0.34	−0.01	0.90***	0.76***	0.74***	0.89***	0.95***	0.92***	0.67***	0.87***
Grain filling duration (GFD)	−0.70***	−0.59***	−0.40*	0.01	0.72***	0.62***	0.63***	0.67***	0.74***	0.71***	0.54***	0.64***

*ETa, actual evapotranspiration; T, actual transpiration; H, estimated plant height*.

***P < 0.001; **P < 0.01; *P < 0.05.

On the other hand, the correlation coefficients calculated for each irrigation treatment separately showed a completely different picture. The number of statistically significant associations between remotely-sensed traits and yield-related traits was much larger for the 50 and 100%ETc treatments than for the rainfed treatment (Table 5). The largest r value obtained for the non-irrigated treatment corresponded to the relationship between LAI and PH during the flight carried out on April 4th (*r* = 0.65, *P* < 0.01), but this relationship was not confirmed in the subsequent image acquisition dates. ETa was negatively and significantly associated with NGS on the first and second image acquisition dates, but this relationship was not significant on the third date (Table 5). Moreover, positive and significant correlation coefficients appeared between ETa and PH on the first and third dates, but not the second. For the 50%ETc treatment, significant correlation coefficients were found for all yield-related traits on at least one image acquisition event, with the exception of the NSm^2^ and GFD which were not associated with any remotely-sensed estimated trait on any date (Table 5). Predicted plant height (H) was significantly and negatively correlated with NGS and positively with GFR on the three image acquisition dates and T was significantly correlated with DH and DA on the second and third dates. No significant relationships were found between remotely-sensed traits assessed at jointing and yield-related traits in the 100%ETc treatment, but H estimated on the second and third dates was significantly related with NGS, TKW, GFR, and PH (Table 5). ETa was positively associated with PH, DH, and DA but negatively with GFD on the second and third dates. GFD was significantly and negatively correlated with the four remote sensing traits on the third date, and with ETa and LAI on the second.

**Table 5:** Significant (*P* < 0.05) Pearson correlation coefficients (r) for the relationships between traits assessed through remote sensing and yield, yield-related traits and crop phenology for each irrigation treatment and each image acquisition event (*n* = 19).

**Trait**	**April 4th**				**April 30th**				**May 22nd**			
	**ET_**a**_**	**T**	**H**	**LAI**	**ET_**a**_**	**T**	**H**	**LAI**	**ET_**a**_**	**T**	**H**	**LAI**
**Irrigation treatment: 100%ETc**												
Number of spikes/m^2^ (NSm^2^)					0.50*					0.48*		
Number of grains per spike (NGS)							−0.53*				−0.69**	
Thousand kernel weight (TKW)							0.63**				0.65**	
Grain filling rate (GFR)							0.59**				0.71***	
Plant height (PH)					0.51*		0.72***		0.59**		0.79***	0.57**
Days to heading (DH)					0.57*			0.47*	0.45*	0.56*		
Days to anthesis (DA)					0.65**			0.49*	0.48*	0.58**		
Grain filling duration (GFD)					−0.71***			−0.49*	−0.59**	−0.64**	−0.59**	−0.57*
**Irrigation treatment: 50%ETc**												
Number of grains per spike (NGS)	−0.50*	−0.48*	−0.57*	−0.67**			−0.73***				−0.62**	
Thousand kernel weight (TKW)			0.52*	0.48*			0.63**					
Grain filling rate (GFR)			0.49*	0.51*			0.59**				0.53*	
Plant height (PH)											0.52*	
Days to heading (DH)						0.63**		0.48*	0.52*	0.58**		
Days to anthesis (DA)						0.58**		0.47*	0.48*	0.58**		
Days to maturity (DM)					0.52*				0.70***	0.55*		
**Rainfed**												
Number of spikes/m^2^ (NSm^2^)									0.59**			
Number of grains per spike (NGS)	−0.57*				−0.56*	−0.46*						
Thousand kernel weight (TKW)							0.46*					
Plant height (PH)	0.50*			0.65**		0.54*			0.54*			
Days to heading (DH)									0.59**			
Days to maturity (DM)								0.63**				
Grain filling duration (GFD)								0.55*				

*Traits with nonsignificant r values for any flight have been omitted. ET_a_, actual evapotranspiration; T, actual transpiration; H, estimated plant height*.

***P < 0.001; **P < 0.01 *P < 0.05.

### Assessment of Genotypic Differences

Figure 7 shows that the largest difference between genotypes for the remotely-sensed traits was recorded in the third flight. The comparison of genotypic values for each trait and irrigation treatment for that flight event showed that, although genotypes differed in their yield at each irrigation treatment, the discrimination power of the remotely-sensed traits varied depending on the water available for the crop (Table 6). Genotypic differences were not statistically significant for H in the rainfed treatment in which significant differences were obtained for LAI, Eta, and T. Genotypes did not differ in ETa and T in either of the irrigated treatments and in LAI in the 50%ETc treatment. In agreement with the positive relationships between yield and either LAI, Eta, and T (Figure 8), the genotypes that reached the highest yields in the 100%ETc treatment (Euroduro, Anvergur, Grador, and others shown in Table 6) tended to have superior values for these three traits, while the lowest yielding ones showed low values for them. However, this trend was not observed for the 50%ETc treatment, where the significant differences detected for yield between the Sculptur and Claudio cultivars, which obtained the highest yields, and the Don Sebastian cultivar, which gave the least yield, were not associated with specific values of the remotely-sensed traits. Even though the rainfed treatment had the highest discrimination power between cultivars, genotypic differences in remotely-sensed traits were also independent of yield variations.

**Table 6:** Comparison of estimated plant height (H), leaf area index (LAI), actual evapotranspiration (ETa), actual transpiration (T), and yield in each genotype on May 22nd (grain filling).

**100%ETc**						**50%ETc**						**Rainfed**					
**Genotype**	**H**	**LAI**	**ETa**	**T**	**Yield**	**Genotype**	**H**	**LAI**	**ETa**	**T**	**Yield**	**Genotype**	**H**	**LAI**	**ETa**	**T**	**Yield**
	**(m)**		**(mm/day)**	**(mm/day)**	**(t/ha)**		**(m)**		**(mm/day)**	**(mm/day)**	**(t/ha)**		**(m)**		**(mm/day)**	**(mm/day)**	**(t/ha)**
EURODURO	0.94^a^	4.08^abc^	7.45	5.63	10.45^a^	CLAUDIO	0.86^abc^	2.88	6.00	5.01	8.47^a^	SCULPTUR	0.61	1.19^ab^	3.85^ab^	2.42^ab^	5.97^a^
ANVERGUR	0.83^bcde^	4.18^abc^	7.45	5.74	10.01^ab^	SCULPTUR	0.80a^bc^	3.00	5.97	5.25	8.27^a^	CLAUDIO	0.65	1.02^ab^	3.83^ab^	2.08^ab^	5.40^ab^
GRADOR	0.92^ab^	3.74^abc^	7.35	5.45	9.98^ab^	ATHORIS	0.78^bc^	2.50	5.23	4.26	8.05^ab^	IBERUS	0.59	0.98^ab^	3.75^ab^	1.99^ab^	5.21^abc^
07D057D4fba	0.84^abcde^	3.78^abc^	7.32	5.52	9.95^abc^	09D066D8cab	0.93^a^	2.81	5.60	4.63	7.90^ab^	ANVERGUR	0.59	1.06^ab^	3.68^ab^	2.19^ab^	5.19^abc^
BURGOS	0.87^abcd^	4.43^a^	7.46	5.92	9.75^abc^	ANVERGUR	0.81^abc^	2.77	5.20	4.49	7.87^ab^	CALERO	0.49	0.78^b^	3.62^ab^	1.58^b^	5.13^abc^
CLAUDIO	0.86^abcde^	4.14^abc^	7.41	5.60	9.52^abcd^	EURODURO	0.90^ab^	2.35	5.43	4.30	7.85^ab^	DON RICARDO	0.70	1.16^ab^	3.82^ab^	2.38^ab^	5.11^abc^
ATHORIS	0.78^def^	3.76^abc^	7.26	5.61	9.45^abcd^	07D057D4fba	0.86^abc^	2.64	5.75	4.65	7.79^ab^	BURGOS	0.54	1.07^ab^	3.91^ab^	2.16^ab^	5.08^abc^
08D010D10cab	0.88^abcd^	4.07^abc^	7.13	5.55	9.39^abcd^	GRADOR	0.86^abc^	2.18	5.40	4.00	7.75^ab^	ATHORIS	0.64	0.96^ab^	3.72^ab^	1.98^ab^	5.03^abcd^
09D066D8cab	0.91^abc^	3.91^abc^	7.38	5.47	9.21^abcd^	CARPIO	0.87^abc^	2.60	5.33	4.51	7.75^ab^	08D010D10cab	0.64	1.16^ab^	4.03^ab^	2.34^ab^	4.88^bcde^
CARPIO	0.86^abcde^	3.78^abc^	7.28	5.60	9.20^abcd^	IBERUS	0.81^abc^	2.87	6.18	4.95	7.62^ab^	EURODURO	0.62	0.97^ab^	3.72^ab^	1.99^ab^	4.87^bcde^
SCULPTUR	0.81^cdef^	3.99^abc^	7.06	5.55	9.16^abcd^	08D010D10cab	0.85^abc^	2.69	5.33	4.59	7.59^ab^	07D057D4fba	0.50	0.91^b^	3.70^ab^	1.85^ab^	4.85^bcde^
09D069D1dcf	0.88^abcd^	3.73^abc^	6.70	5.25	9.09^abcde^	09D069D1dcf	0.86^abc^	2.79	5.45	4.95	7.59^ab^	GRADOR	0.52	0.77^b^	3.56^ab^	1.57^b^	4.65^bcde^
DON RICARDO	0.92^ab^	3.53^abc^	7.16	5.48	9.02^abcde^	CALERO	0.73^c^	2.52	5.82	4.47	7.58^ab^	09D069D1dcf	0.53	1.02^ab^	3.78^ab^	2.06^ab^	4.62^bcde^
IBERUS	0.79^def^	4.47^a^	7.46	5.95	8.97^abcde^	BURGOS	0.84^abc^	3.12	5.60	4.87	7.54^ab^	09D066D8cab	0.55	0.92^b^	3.87^ab^	1.87^ab^	4.36^bcde^
DON SEBASTIAN	0.92^ab^	4.26^ab^	7.16	5.58	8.83^abcde^	DON RICARDO	0.89^ab^	2.57	5.29	4.40	7.51^ab^	EUNOBLE	0.60	1.06^ab^	3.85^ab^	2.17^ab^	4.30^cde^
EUNOBLE	0.89^abcd^	3.31^abc^	6.96	5.24	8.31^bcde^	05D278D1be	0.88^abc^	2.28	5.18	4.14	7.01^ab^	CARPIO	0.57	0.98^ab^	3.70^ab^	2.01^ab^	4.29^cde^
CALERO	0.70^f^	2.98^c^	6.48	4.87	8.10^cde^	TUSSUR	0.73^c^	2.47	5.34	4.18	6.64^ab^	TUSSUR	0.48	0.83^b^	3.51^b^	1.71^b^	4.23^cde^
05D278D1be	0.83^abcde^	3.11^bc^	6.85	4.93	7.82^de^	EUNOBLE	0.86^abc^	2.24	5.21	3.93	6.48^ab^	05D278D1be	0.74	0.87^b^	3.65^ab^	1.81^b^	4.00^de^
TUSSUR	0.76^ef^	3.01^c^	6.46	4.91	7.28^e^	DON SEBASTIAN	0.93^a^	3.21	5.69	4.83	5.91^b^	DON SEBASTIAN	0.61	1.48^a^	4.24^a^	2.92^a^	3.91^e^

*Genotypes ordered by yield. Different letters in the same column mean significant differences between genotypes at p ≤ 0.05 using Tukey's honest significant difference test*.

## Discussion

The present study provided a quantifiable assessment of UAV imagery for the purpose of obtaining an accurate estimation of the agronomic performance of durum wheat from the field phenotyping of 19 durum wheat genotypes grown under three contrasting water regimes. The proposed method employs the TSEB model to estimate differences between irrigation treatments and genotypes in actual crop evapotranspiration (ETa) and transpiration (T). The biophysical parameters of the vegetation, such as LAI and canopy height (H) were respectively estimated through spectral vegetation indices and photogrammetry. The feasibility of using this methodology for high-throughput field phenotyping has been demonstrated, since it is robust, repeatable and time, and cost efficient compared with measurements made at ground level.

### Effect of Water Availability on Durum Wheat Field Performance

The experimental site is representative of the Mediterranean climate, with a long-term mean temperature of 10.4°C and average rainfall of 248 mm from November to June. This mean temperature was recorded for the 2018–2019 growing season, but rainfall was slightly lower than average. This water scarcity allowed the testing of three contrasting irrigation treatments. Results indicate that a water input of 450 mm (rainfall + irrigation), most of which was supplied during the spring, was enough to cover all the evapotranspiration needs of the durum wheat crop (Figure 5). A reduction of 36.7% in the water supplied (285 mm) covered half of these needs (50%ETc), while the non-irrigated treatment (122 mm of rainfall) represented 27.1% of the water needed to meet evapotranspiration needs. The analyses of the effects of water constraints on grain yield revealed that supplying 63.3% (50%ETc) or 27.1% (rainfed treatment) of the water needed to cover the whole crop evapotranspiration needs, resulted in yields that corresponded to 81.7 and 52.0%, respectively, of the yield obtained in the full irrigation treatment (100%ET). Karam et al. (2009) obtained yield decreases between 25 and 28% in rainfed and half-irrigated durum wheat compared with a full-irrigated treatment. These results suggest that durum wheat could be an alternative for irrigated areas with low seasonal water availability, since a reduction of 36.7% in water input decreased yield by only 18.3%, and a water reduction of 72.9% diminished yield by 48.0%. In terms of water productivity (WP), the values were 2.05, 2.64, and 3.93 kg of DM grain/m^3^ of water applied in the 100%ETc, 50%ETc, and rainfed treatments, respectively. These results reflect the efforts made by breeders to improve drought tolerance of modern durum wheat cultivars adapted to drought-prone environments (Araus et al., 2003), where yield differences between drought-tolerant and drought-sensitive ideotypes are evident (Senapati et al., 2019).

The 18.3% yield reduction observed in the 50%ETc treatment when compared with the 100%ETc was a consequence of decreases of 15.0% in NGS and 3.8% in TKW, as the NSm^2^ was not affected. In addition, the 48.0% yield decrease of the rainfed treatment in comparison with the fully-irrigated one was due to a reduction of 33.7% in NGS, 9.8% in NSm^2^, and 7.7% in TKW. In this study, the larger LAI values estimated for the rainfed treatment at the jointing stage suggest that the tiller number was probably not strongly affected by drought, which is in agreement with the low reduction of NSm^2^ observed in the rainfed treatment. Our results agree with the assumption that NGS is typically the yield component that is most sensitive to drought stress due to severe competition for nutrients during stem elongation (Richards et al., 2001; Kilic and Yagbasanlar, 2010; Liu et al., 2015). Decreases in the NGS from 12.4 to 58.7% have been found in durum wheat under drought stress compared to well-irrigated conditions (Vahamidis et al., 2019).

When compared with the fully-irrigated treatment, the cycle shortening observed in this study ranged between 3 days (2.2%) for DH in the 50%ETc treatment to 13 days (7.1%) for DM in the rainfed treatment. Similar reductions have been reported in the literature (Liu et al., 2015; Varga et al., 2015). The reductions observed in DA and DM in the 50%ETc and rainfed treatments suggest that water stress likely accelerated leaf senescence, which is a common response to water shortage (Ihsan et al., 2016; Pour-Aboughadareh et al., 2020). In relative terms, the greatest shortening was observed in GFD (up to 12.5%), which could not be compensated by the increase of 5.7% in GFR. Decreases of 14% in the duration of grain filling have been reported previously in durum wheat subjected to pre-anthesis drought (Liu et al., 2015). It is well-known that the reduced grain-filling period directly affects grain number and grain size, which largely accounts for the decrease in wheat yields (Dolferus et al., 2011). Plant height decreased 6.5 and 12.0% in the 50%ETc and rainfed treatments, respectively, which is in agreement with the biomass reduction caused by drought shown by previous studies (Pour-Aboughadareh et al., 2020 and references herein).

### Predicted vs. Observed Traits

Spectral vegetation indices (VI) assessed from ground level though field spectrometry have been widely used to estimate several wheat traits such as growth status, biomass, yield, or photosynthesis (Aparicio et al., 2000, 2002; Magney et al., 2016). Moreover, UAV-derived VI [e.g., NDVI, soil adjusted VI (SAVI) and optimized soil adjusted VI (OSAVI)] have also been used to estimate the same traits (Yue et al., 2019; Marino and Alvino, 2020), but with the advantages over field spectrometry of generating surface maps in real time, higher flexibility and more convenient operation for estimating plant traits from large numbers of plots at a time (Lelong et al., 2008; Maimaitijiang et al., 2017). UAV high-resolution VI may detect changes of plant status, thus helping to improve crop monitoring, nitrogen management, and crop yield estimation (Cabrera-Bosquet et al., 2011). However, the relationships between existing VI and biophysical parameters of the vegetation, such as LAI, usually generate an error, in part because some of them saturate at medium-to-high canopy covers, are sensitive to the chlorophyll content or to internal factors such as canopy geometry, leaf and soil optical properties (Baret and Guyot, 1990; Zhou et al., 2017). In this study, the MTVI_2_ showed a linear relationship with LAI, with an *R*^2^ of 0.78 (Figure 6A). This positive linear regression could be attributable to the MTVI_2_ having a center wavelength located at the red-edge region (717 nm), which is mainly influenced by the plant's structural characteristics and chlorophyll content (Guyot et al., 1992; Yao et al., 2017; He et al., 2020). This suggests that the MTVI_2_ is not as sensitive to canopy structure as other indices which only use bands at the red and near-infrared regions.

Crop height (H) estimates through photogrammetry were regularly underestimated by 0.18 ± 0.05 m. According to Lechner et al. (2009) and Hengl (2006), the image spatial resolution has to be at least half of the size of the target object to be accurately discriminated though photogrammetric analysis. Therefore, it is possible that the low leaf width of durum wheat, similar to the pixel size (0.02 m), provoked this systematic underestimation (Figure 6C). Probably, increasing the number of images acquired from different viewing angles, with a higher overlap, could help to improve H estimates. However, these results are in agreement with those obtained in previous studies at the same spatial resolution in wheat (Holman et al., 2016; Demir et al., 2018), and olive trees (Caruso et al., 2019). Since plant height is one of the necessary ancillary data of the TSEB model, a precise estimation of H is essential to assess plant evapotranspiration.

Estimates of canopy transpiration were validated against leaf transpiration measurements (Figure 6D). Although remotely-sensed estimates of T were higher than the measured ones, the relationship had an *R*^2^ of 0.50. The higher T rates assessed through remote sensing were because they were calculated at plot level, whereas the others were calculated only at leaf level. Differences could also be attributable to the likelihood that the partitioning of ET into T and E contains a substantial bias error.

### Relationships Between Traits Assessed From Remote Imagery and Agronomic Traits

The sign of the correlation coefficients between remotely-sensed traits and most of the agronomic characteristics were negative in the first flight and positive in the subsequent ones. This was due to the high initial vegetative growth of the plots subjected to the rainfed treatment, as revealed by the LAI, ETa, and T values shown in Table 3, which likely reflects the effect of soil variations on the growth of seedlings. It is probable that soil water holding capacity was higher in the area were the rainfed treatment was located. As the season evolved, this trend was reversed and the fully-irrigated treatment showed the highest evapotranspiration rates which previous studies have associated with higher stomatal conductance and photosynthetic rates (Fischer et al., 1998). The highest ETa and T values observed in the 100%ET treatment are in agreement with high yielding wheat cultivars showing higher rates of transpiration (Shimshi and Ephrat, 1975; Reynolds et al., 1994) and with the strong association existing between T and LAI (Blum, 2011).

When the analyses of the relationships between grain yield and the four traits assessed from remote sensing images (H, ETa, T, and LAI) were conducted using the aggregated data of the three irrigation treatments for each flight event, the results clearly show that forecasts were much more accurate at anthesis and grain filling than at jointing (Figure 7). Except for H, the correlation coefficients were in general slightly higher in the third flight (May 22nd) than in the second (April 30th), thus suggesting that yield predictions were more accurate when images were captured during grain filling than around anthesis. A lower correlation in LAI was observed at flowering (2nd flight date, 147DAS) in comparison to grain filling (3rd flight date, 169DAS). This can probably be explained by an early senescence reached in some of the genotypes (Table 1). Greater variability may explain an increase in the correlation with respect to the second flight date. The analyses conducted for the yield-related traits confirmed that measurements at advanced crop stages were better, as demonstrated in previous studies (Hassan et al., 2018). This was an expected result, as only the potential number of spikes and spikelets per spike are defined at jointing (Simane et al., 1993), while grain setting, grain weight and final yield are determined in subsequent developmental stages (Giunta et al., 1993). NGS, PH, DH, DA, and DM and GFD could be properly assessed through remotely acquired estimates of ETa, T, H, and LAI during grain filling (Table 4). The highest *R*^2^ to estimate yield components was observed in ETa rather than with T. It is crucial for ET partitioning to retrieve reliable estimates of canopy and soil temperatures, net radiation, and aerodynamic roughness, with the latter usually obtained from vegetation structural parameters. Therefore, any bias in those estimates could be a source of error when attempting to obtain accurate estimates of T. In addition, the higher range of variability of ETa values in comparison to T contributed to obtaining the highest *R*^2^ when it was regressed with yield. This is because ETa also uses the soil temperature (T_soil_) of each individual plot and irrigation treatment, with important differences in T_soil_ between irrigated and rainfed plots. On the other hand, predicted plant height (H) was also a good estimator of DH, DA, DM, GFD, PH, and TKW at anthesis, although the values were slightly lower in comparison to the evapotranspiration components. While in this study H was estimated from photogrammetry using multispectral imagery, the advantages of using H instead of ET estimates include the need for fewer inputs, and the lower cost and amount of time needed. Plant height is an essential trait in wheat as it determines the architecture of the plant canopy and has a strong effect on grain number, harvest index and final grain yield (Maccaferri et al., 2008; Liu et al., 2015). The relationships between plant height and yield are environmentally dependent as positive associations have been reported under optimal water conditions and negative associations in water stress environments (Royo et al., 2008; Dogan, 2009; Talebi et al., 2010). Plant height has been proposed as a potential indicator of tolerance to drought stress under Mediterranean conditions (Liu et al., 2015).

Although the *R*^2^ of the relationships between the assessed parameters and NSm^2^ were significant, the weaker relationships obtained for this trait suggest that it cannot be considered a yield component that it is possible to properly estimate through remote imagery. GFR could not be assessed through any remotely-sensed trait, even when considering the aggregated data of the three irrigation treatments (Table 4). However, when the relationships between remotely-sensed estimated traits and yield-related traits were analyzed individually for each irrigation treatment, results showed higher accuracy in the irrigated treatments than in the rainfed one (Table 5). Although some *r* values obtained from the regressions between remotely-sensed estimated traits and the agronomic ones were significant under rainfed conditions, they did not show consistency among related traits nor across image acquisition dates. This suggests that they could be more casual than causal, and therefore do not demonstrate enough reliability to be recommended for accurate field assessments. This was probably related, as discussed previously, with the wider range of values observed for most traits in the irrigated treatments when compared with the rainfed treatment, as shown in Table 1, which increased the predictability of remote sensing imagery. The comparison of the number of significant correlation coefficients obtained in each irrigated treatment in the second and third flights and their values revealed that assessments made in the 100%ETc treatment showed more significant correlation coefficients and with higher values than the ones made in the 50%ETc treatment (Table 5). As regards the growth stage most appropriate for predicting yield-related traits, the number of significant correlation coefficients indicated that, as in the case of yield, the second (at anthesis) and third (during grain filling) image acquisition dates were the most suitable, but a higher number of positive associations were found for the third flight.

In relation to the agronomic traits that can be properly assessed by remote sensing imagery, the negative and significant correlation coefficients between H and NGS in the two irrigated treatments and the two later flights (*r* values from −0.53 to −0.73) suggest a causal and consistent association. Similarly, H showed a positive and significant association with GFR in the same four cases, with r values ranging from 0.59 to 0.71, thus indicating a good predictive value. The analysis of the relationships between predicted plant height and TKW showed less consistent results given that the correlation coefficient obtained for the third flight in the 50%ETc treatment was not statistically significant. DH and DA were among the phenological characteristics that were most consistently related with remotely-sensed traits, with ETa being the best predictor for them, mostly during the third flight. GFD was negatively and consistently related with the four remotely-sensed traits estimated from images acquired during the grain filling stage in the fully-irrigated treatment.

Remote sensing imagery has been widely used to assess yield-related traits under a wide range of phenotypical variations (Aparicio et al., 2002; Haghighattalab et al., 2016; Caruso et al., 2019). In the current study, when data of the three irrigation treatments were analyzed together, the yield ranged between 3,905 and 10,446 kg/ha. Previous studies showed that this very wide range of variability is exceptional for durum wheat genotypes grown in the same site where this study was carried out when subjected to a common agronomic management, under both irrigated and rainfed conditions (Aparicio et al., 2000). For this reason, we also decided to assess the suitability of remotely-sensed estimated traits within each irrigation treatment, given that such homogeneous environmental conditions are more representative of real-world cropping systems. In this case, the results showed that yield could only be properly forecasted in the 100%ETc treatment (Figure 8). The lack of water restrictions probably allowed the genotypes to express their potentialities, thus maximizing phenotypic differences as shown by the wider range of yields observed in the 100%ETc treatment (3,172 kg/ha) compared with the 50%ETc (2,559 kg/ha) and the rainfed treatment (2,067 kg/ha). Previous studies have also demonstrated that the capacity of spectral reflectance indices to forecast durum wheat grain yield was higher in locations where genotypes reached potential yields (Royo et al., 2003). Under full irrigation conditions (100%ETc), the results of this study also indicate that ETa was the best predictor of yield, particularly when image acquisition was performed around anthesis or during grain filling. On both dates, it accounted for about 68% of yield variations (Figure 8).

### Capacity of Remotely-Sensed Traits to Discriminate Among Genotypes

The analysis of the data for each genotype provided by the remotely-sensed traits assessed during grain filling gave a wide range of values for all of them. However, in some cases the differences were not wide enough to be statistically significant (Table 6). For LAI, ETa, and T, the highest statistical significance was obtained in the rainfed treatment. Although the pattern behind these results was not totally clear, the relatively wider range of values recorded in the rainfed treatment when compared with the irrigated ones could partially explain these differences. Though the absolute values of LAI, ETa, and T were greater in the irrigated treatments than in the rainfed one, in relative terms the differences between the values of the genotypes showing the highest and the lowest value for each trait were largest in the latter. For instance, in the rainfed treatment, the *T* value of cv. Don Sebastian (2.92 mm/day) was 86% superior to that of cv. Grador, which showed the lowest estimate (1.57 mm/day). This relative difference, which was superior to that obtained in the 100%ETc (17.9%) and the 50%ETc (33.5%) treatments was large enough to prove statistically that these two genotypes differed for this trait. Similarly, the relative wider variations among the extreme values for ETa and LAI obtained in the rainfed treatment than in the irrigated ones support the differences obtained in statistical significances. In the case of H, the lack of differences between genotypes in the rainfed treatment could not be attributed to the same reason, as the relative difference in H values was 54%, larger than that observed in the irrigated treatments (23.7 and 27.4% in 100 and 50%ETc, respectively) where statistically significant differences were detected. In this case, the reason could likely be the low H values in the rainfed treatment resulting from the short plants, associated to drought environments (Madec et al., 2017), and the underestimation of actual plant height occasioned by the methodology employed which was in accordance with previous studies (Holman et al., 2016).

According to other studies that related durum wheat transpiration and yield (Medina et al., 2019), the genotypes with the highest yields in the fully-irrigated treatment showed superior LAI, ETa, and T values. A high LAI in durum wheat genotypes at the milk-grain growth stage denotes a delay of leaf senescence after anthesis, a characteristic that has been positively related with grain yield (Borojevic et al., 1980), thus underlining its importance as a grain yield determining feature. The high values for ETa and T in high-yielding genotypes are in agreement with the positive associations found between T and both leaf area and biomass in wheat grown in well-watered environments (Blum, 2011).

## Conclusions

This study shows the feasibility of using the two-source energy balance (TSEB) with very high resolution imagery to assess differences in the evapotranspiration components of a durum wheat panel. For this purpose, biophysical parameters of the vegetation were successfully estimated from multispectral imagery. Plant height and LAI estimates gave RMSE values of 0.18 m and 0.63, respectively. Significant differences in durum wheat yield and yield components were observed between irrigation treatments. The 50%ETc and rainfed treatments accounted for respective yield reductions of 18.3 and 48.0% in comparison with the treatment that met all crop water requirements (100%ETc). ETa was the remotely-sensed parameter that, when estimated either at anthesis or during grain filling, showed a positive relationship and the highest *R*^2^ with yield, DH, DA, and GFD. When data were analyzed individually for each irrigation treatment, consistent and positive associations were found between ETa and yield, DH and DA and negative associations with GFD in the 100%ETc treatment, but not in the other treatments. The remotely-sensed traits that were assessed were able to discriminate among genotypes, but the significance of the differences depended on the irrigation treatment. As a conclusion, this study demonstrates that remotely-sensed estimates of ETa through the TSEB model are the best predictor of yield components. *R*^2^ values at the grain filling stage were higher in comparison with other remotely-sensed trait estimates such as height, LAI or spectral vegetation indices.

## Data Availability Statement

The raw data supporting the conclusions of this article will be made available by the authors, without undue reservation.

## Author Contributions

CR led the project. DG-C, CR, and JB conceived the manuscript, analyzed the data, and wrote the manuscript. All authors contributed to the article and approved the submitted version.

## Conflict of Interest

The authors declare that the research was conducted in the absence of any commercial or financial relationships that could be construed as a potential conflict of interest.

## Abbreviations

ET: evapotranspiration
T: transpiration
H: predicted plant height
PH: observed plant height
DH: days from sowing to heading
DA: days from sowing to anthesis
DM: days from sowing to maturity
DAS: days after sowing
GFD: grain filling duration
NSm^2^: number of spikes/m^2^
NGS: number of grains/spike
TKW: thousand kernel weight
GFR: grain filling rate
GFD: grain filling duration.
