# Supplementary material for: Performance of the Two-Source Energy Balance (TSEB) Model as a Tool for Monitoring the Response of Durum Wheat to Drought by High-Throughput Field Phenotyping
Source: Front Plant Sci. 2021 Apr 16;12:658357. doi: 10.3389/fpls.2021.658357 (PMC8085348; doi:10.3389/fpls.2021.658357)
Supplement: Supplementary file 1 [file Table_1.pdf]

Supplementary Table 1. Genotype means across replications for the assessed traits. Traits estimated through remote sensing: ET_a, actual evapotranspiration; T, actual transpiration; H, plant height; $MTVI_2$, LAI surrogate. Numbers after each trait indicate the image acquisition event: 1, April 4th (jointing); 2, April 30th (anthesis); 3, May 22nd (grain filling). Measured traits at ground level: DH, days to heading; DA, days to anthesis; DM, days to maturity; GFD, grain filling duration; PH, plant height; NSm^2, number of spikes/m^2; NGS, number of grains/spike; TKW, thousand kernel weight; GFR, grain filling rate.

Genotpe	ETa1	T1	H1	$MTVI_2$1	ETa2	T2	H2	$MTVI_2$1	ETa3	T3	H3	$MTVI_2$1	DH	DA	DM	GFD	Yield	PH	NSm2	NGS	TKW	GFR
											Irrigation treatment 100%Etc											
05D278D1be	4.739	2.339	0.380	1.608	6.531	4.241	0.815	3.941	6.848	4.926	0.878	4.095	131	138	181	43	7816	95	369	41.3	57.27	1.33
07D057D4fba	4.946	2.576	0.354	1.476	6.894	4.405	0.721	3.935	7.320	5.523	0.834	3.986	137	145	185	40	9942	95	440	39.3	60.70	1.52
08D010D10cab	4.868	2.458	0.413	1.574	6.909	4.436	0.724	4.569	7.125	5.548	0.895	4.770	136	143	182	39	9390	92	707	37.7	50.73	1.30
09D066D8cab	4.984	2.416	0.369	1.680	6.799	4.306	0.747	4.514	7.376	5.474	0.890	4.908	138	146	185	39	9210	97	533	38.6	59.20	1.53
09D069D1dcf	4.716	2.389	0.409	1.691	6.810	4.502	0.775	4.537	6.697	5.249	0.888	4.012	136	144	184	40	9087	92	542	37.5	59.20	1.49
ANVERGUR	4.681	2.267	0.308	1.565	6.863	4.407	0.757	4.592	7.446	5.736	0.856	4.950	138	147	186	39	10008	92	436	50.1	53.57	1.36
ATHORIS	4.833	2.556	0.349	1.998	6.776	4.547	0.724	4.682	7.259	5.612	0.779	4.521	132	139	181	43	9449	83	480	43.3	53.17	1.25
BURGOS	4.825	2.449	0.407	1.501	6.852	4.609	0.712	4.676	7.459	5.918	0.882	4.911	138	147	184	37	9750	100	653	37.2	60.73	1.63
CALERO	4.705	2.273	0.376	1.518	6.597	4.611	0.617	3.978	6.479	4.871	0.703	2.643	137	143	185	42	8102	82	289	60.3	45.47	1.08
CARPIO	4.671	2.414	0.324	1.615	6.798	4.458	0.757	4.080	7.284	5.602	0.838	4.163	134	141	182	41	9194	93	453	51.5	53.23	1.30
CLAUDIO	5.117	2.676	0.427	2.487	6.884	4.461	0.819	5.101	7.407	5.603	0.899	5.014	136	144	184	40	9515	98	524	40.2	55.43	1.40
DON_RICARDO	4.859	2.544	0.419	2.137	6.746	4.506	0.842	4.505	7.162	5.481	0.928	4.317	135	143	181	38	9022	102	453	42.0	62.37	1.64
DON_SEBASTIAN	5.053	2.404	0.387	1.537	6.932	4.402	0.793	4.996	7.161	5.584	0.940	4.846	136	146	184	39	8830	100	498	34.3	63.17	1.63
EUNOBLE	5.008	2.558	0.355	1.749	6.744	4.385	0.764	4.242	6.955	5.237	0.900	4.183	130	138	180	43	8307	88	471	41.4	51.13	1.20
EURODURO	5.225	2.678	0.390	2.083	6.933	4.394	0.772	4.400	7.450	5.630	0.920	4.564	135	144	183	38	10446	95	742	37.4	55.17	1.44
GRADOR	5.060	2.501	0.412	1.980	6.843	4.324	0.817	4.297	7.349	5.445	0.905	4.208	134	142	182	40	9981	93	338	36.7	64.80	1.61
IBERUS	4.872	2.492	0.389	2.152	6.907	4.553	0.741	5.271	7.465	5.953	0.809	5.410	138	145	185	40	8971	90	538	39.1	50.37	1.25
SCULPTUR	4.985	2.405	0.399	1.556	6.756	4.503	0.684	4.642	7.062	5.553	0.832	4.697	135	142	183	41	9161	85	516	52.0	49.30	1.20
TUSSUR	4.617	2.225	0.340	1.463	6.537	4.407	0.676	3.735	6.457	4.914	0.794	2.484	132	139	182	43	7274	80	529	40.3	55.07	1.28
											Irrigation treatment 50%Etc											
05D278D1be	5.407	2.465	0.391	2.054	6.211	4.316	0.753	3.260	5.184	4.136	0.979	2.949	127	135	175	40	7011	90	462	34.1	55.77	1.38
07D057D4fba	5.415	2.504	0.384	2.225	6.372	4.534	0.786	3.989	5.747	4.652	0.914	3.961	133	140	177	37	7792	87	400	34.3	55.77	1.49
08D010D10cab	5.471	2.680	0.347	2.317	6.230	4.467	0.748	3.694	5.332	4.594	0.899	3.634	131	138	175	37	7594	90	564	30.2	51.13	1.37
09D066D8cab	5.483	2.453	0.406	2.327	6.393	4.396	0.714	3.767	5.604	4.627	1.019	3.314	135	143	178	35	7896	90	556	32.9	56.17	1.62
09D069D1dcf	5.465	2.572	0.388	2.249	6.413	4.580	0.753	3.923	5.447	4.954	0.864	3.376	132	139	176	37	7588	87	609	34.9	53.47	1.45
ANVERGUR	5.258	2.577	0.314	2.177	6.399	4.553	0.742	4.284	5.196	4.495	0.864	3.292	135	142	178	36	7863	90	493	46.9	51.43	1.44
ATHORIS	5.494	2.579	0.339	2.284	6.333	4.530	0.698	3.883	5.234	4.261	0.841	3.962	130	137	175	38	8046	77	529	39.0	51.50	1.36
BURGOS	5.375	2.645	0.411	2.745	6.378	4.677	0.802	4.565	5.603	4.875	0.941	3.927	136	143	177	35	7539	88	556	32.8	57.97	1.67
CALERO	5.238	2.398	0.339	1.986	6.426	4.440	0.624	3.729	5.820	4.468	0.709	2.915	133	140	180	40	7578	80	498	53.9	44.87	1.11
CARPIO	5.096	2.421	0.384	2.077	6.452	4.478	0.744	3.954	5.329	4.509	0.958	3.008	130	137	177	39	7749	88	542	41.0	52.73	1.34
CLAUDIO	5.533	2.630	0.375	2.500	6.720	4.624	0.754	4.406	6.003	5.005	0.863	3.376	133	140	178	38	8469	95	600	35.3	51.60	1.36
DON_RICARDO	5.299	2.605	0.459	2.514	6.254	4.387	0.839	4.117	5.294	4.401	0.928	3.836	131	138	174	37	7512	85	387	33.0	59.37	1.62
DON_SEBASTIAN	5.462	2.661	0.426	2.793	6.505	4.578	0.844	4.497	5.686	4.834	0.946	4.116	131	138	177	39	5910	95	569	23.6	61.30	1.59
EUNOBLE	5.573	2.583	0.380	2.492	6.451	4.392	0.781	3.994	5.208	3.928	0.883	2.866	128	135	175	40	6478	83	591	29.4	50.87	1.28
EURODURO	5.482	2.642	0.357	2.490	6.337	4.425	0.734	3.883	5.431	4.302	0.915	2.660	133	139	176	37	7844	88	498	30.1	54.83	1.50
GRADOR	5.341	2.478	0.377	2.354	6.456	4.358	0.735	3.501	5.397	3.996	0.776	2.064	130	137	175	38	7754	90	564	36.1	61.23	1.61
IBERUS	5.533	2.603	0.413	2.405	6.521	4.643	0.761	4.241	6.177	4.948	0.899	4.151	134	141	178	37	7615	82	627	31.9	48.60	1.30
SCULPTUR	5.614	2.697	0.392	2.426	6.615	4.508	0.736	4.313	5.966	5.252	0.827	3.816	131	139	178	39	8268	85	440	41.7	48.10	1.22
TUSSUR	5.164	2.507	0.362	2.417	6.283	4.358	0.718	4.149	5.344	4.183	0.778	3.749	130	137	177	40	6641	73	458	38.8	54.30	1.35
											Rainfed											
05D278D1be	5.622	2.605	0.409	1.591	4.638	3.518	0.320	1.927	3.649	1.806	0.707	0.756	125	134	167	34	4001	85	404	30.1	48.80	1.45
07D057D4fba	5.618	2.687	0.325	2.353	4.758	3.679	0.394	2.617	3.698	1.852	0.392	0.823	130	137	174	37	4848	82	489	26.1	54.33	1.48
08D010D10cab	5.803	2.634	0.475	1.250	4.982	4.017	0.396	2.301	4.033	2.336	0.705	1.176	130	136	171	35	4875	82	467	26.6	47.90	1.36
09D066D8cab	5.878	2.694	0.407	1.944	4.725	3.543	0.399	2.391	3.867	1.866	0.385	1.014	130	136	168	32	4363	82	476	27.1	52.33	1.64
09D069D1dcf	5.708	2.760	0.373	2.074	4.753	3.659	0.357	2.549	3.783	2.064	0.380	1.286	129	135	169	34	4617	78	564	25.6	51.17	1.50
ANVERGUR	5.172	2.518	0.367	1.092	4.503	3.637	0.356	2.551	3.682	2.191	0.687	1.492	128	137	173	36	5194	78	413	38.8	49.80	1.38
ATHORIS	5.327	2.659	0.419	1.197	4.514	3.506	0.345	2.142	3.717	1.977	0.645	1.171	126	134	169	35	5030	78	440	33.3	51.97	1.48
BURGOS	5.594	2.588	0.573	1.160	4.742	3.574	0.490	2.330	3.913	2.165	0.525	1.500	129	137	169	33	5086	85	524	24.0	52.03	1.59
CALERO	5.120	2.488	0.331	1.780	4.255	3.212	0.395	2.251	3.620	1.583	0.406	0.773	128	136	172	36	5126	75	391	35.4	45.13	1.24
CARPIO	5.510	2.593	0.367	1.744	4.568	3.711	0.351	2.630	3.702	2.009	0.544	1.245	127	134	169	35	4292	83	404	31.9	49.17	1.39
CLAUDIO	5.745	2.794	0.408	2.703	4.962	4.029	0.551	3.226	3.829	2.080	0.650	1.253	128	136	173	37	5395	83	476	29.8	51.60	1.41
DON_RICARDO	5.833	2.635	0.464	1.365	4.691	3.694	0.671	2.481	3.821	2.382	0.750	1.677	127	134	169	35	5111	82	356	28.8	56.43	1.60
DON_SEBASTIAN	5.975	2.741	0.378	2.469	5.181	4.075	0.668	3.082	4.241	2.923	0.665	1.618	130	136	172	36	3905	92	591	15.3	58.43	1.62
EUNOBLE	6.046	2.814	0.436	2.636	5.288	4.193	0.545	3.142	3.845	2.165	0.614	1.269	125	133	172	38	4301	83	418	23.7	51.03	1.33
EURODURO	5.596	2.654	0.400	1.165	4.652	3.704	0.319	2.011	3.723	1.993	0.428	1.219	127	135	167	32	4869	82	467	21.9	52.87	1.64
GRADOR	5.718	2.673	0.429	2.270	4.918	3.747	0.396	2.543	3.561	1.566	0.472	0.442	125	134	168	34	4651	82	413	23.7	57.13	1.68
IBERUS	6.095	2.901	0.381	2.568	4.975	4.178	0.435	2.715	3.752	1.993	0.563	0.757	129	137	171	34	5208	83	458	24.6	47.03	1.38
SCULPTUR	5.803	2.690	0.428	2.032	4.687	3.700	0.556	2.571	3.849	2.424	0.635	1.312	127	135	171	36	5972	72	413	37.6	49.87	1.39
TUSSUR	5.344	2.535	0.425	1.071	4.210	3.407	0.357	2.104	3.512	1.711	0.566	0.791	124	133	169	36	4225	75	431	25.0	50.77	1.41
